# Effects of roadside memorials on drivers’ risk perception and eye movements

**DOI:** 10.1186/s41235-019-0184-1

**Published:** 2019-08-14

**Authors:** Vanessa Beanland, Rachael A. Wynne

**Affiliations:** 1 Centre for Human Factors and Sociotechnical Systems, University of the Sunshine Coast, Sippy Downs, QLD Australia; 2 Department of Psychology, University of Otago, PO Box 56, Dunedin, 9054 New Zealand

**Keywords:** Visual attention, Driver behavior, Road safety, Roadside memorials

## Abstract

**Abstract:**

Road crashes are a leading cause of death worldwide. In many countries, it is common to see spontaneous roadside memorials constructed in response to road fatalities. These memorials are controversial and are explicitly banned in many jurisdictions. Advocates argue that the presence of memorials improves safety by making other drivers aware of an especially dangerous road where others have died, whereas opponents argue that they are distracting and decrease safety by diverting drivers’ attention away from the road. However, there has been almost no research examining the effects of roadside memorials on road user behavior and safety. In this study, 40 drivers viewed videos of road scenes with and without memorials, to examine how the presence of roadside memorials influences drivers’ attentional allocation (indicated by eye movements to the roadside area) and safety-related behaviors (indicated by perceived risk ratings and preferred travel speeds for the road). The findings indicate memorials do capture visual attention, as participants were more likely to fixate on memorials compared with a comparison object placed on the roadside. However, fixations on the memorials, and to the roadside area in general, were relatively brief. The presence of memorials did not affect perceived risk and did not produce a clear systematic effect on preferred travel speed. Nearly all drivers in our study supported permitting roadside memorials, but a small number strongly opposed memorials on the belief they are distracting and/or distressing.

**Preregistration details:**

This study was preregistered with *Cognitive Research: Principles and Implications* and received in-principle acceptance on 4 March 2018. The preregistered protocol is available here: 10.6084/m9.figshare.6181937.

**Electronic supplementary material:**

The online version of this article (10.1186/s41235-019-0184-1) contains supplementary material, which is available to authorized users.

## Significance

Many cultures have a tradition of establishing spontaneous roadside memorials following a fatal road crash. These memorials mark the place where someone died prematurely, which allows their loved ones to publicly mourn the death and may also serve to warn other drivers of dangerous road conditions. Government policies around roadside memorials are mixed, with many authorities believing they distract drivers. Despite this, there has been almost no scientific research examining whether roadside memorials are distracting or whether they influence safety-related behaviors. We conducted a study examining whether roadside memorials divert drivers’ attention away from the road (i.e., by recording their eye movements while they viewed videos of road scenes) and whether it affects their judgements of how safe the road is and what speed they should adopt. The findings indicate that although memorials may capture drivers’ attention, they do not have a large impact on behavior and are unlikely to have a major impact on road safety (either positive or negative). Further, most drivers who participated believed roadside memorials should be permitted, with a minority strongly objecting to their presence. However, several participants suggested the design of memorials should be regulated to ensure they are safely located and not too eye-catching.

## Introduction

Road crashes are a leading cause of death worldwide, with an estimated 1.25 million fatalities annually (World Health Organization, 2015). In many countries, it is common to see spontaneous roadside memorials constructed in response to fatalities (Clark & Cheshire, 2003). These memorials are typically placed on or near the roadside to commemorate the location where an individual died (Collins & Rhine, 2003). They vary in appearance but often feature motifs such as crosses, flowers, the deceased’s name and/or their date of death (Clark & Cheshire, 2003; Hartig & Dunn, 1998). Studies have indicated that roadside memorials disproportionately memorialize young drivers (Clark & Cheshire, 2003; Collins & Rhine, 2003), especially young men (Hartig & Dunn, 1998), reflecting the fact that young drivers are over-represented in the road toll (Bureau of Infrastructure, Transport and Regional Economics, 2016). The exact prevalence of roadside memorials is unknown, but research found that in the Hunter Valley region of New South Wales, Australia, approximately 20% of fatal crash sites were memorialized and 95% of drivers have seen a roadside memorial at some point (Hartig & Dunn, 1998).

Roadside memorials are often controversial: many authorities believe they have the potential to distract drivers (Churchill & Tay, 2008) but others have incorporated memorials into road safety campaigns (Clark, 2004). Surveys in the USA and Canada have revealed substantial diversity in the existence and nature of government policies on roadside memorials, ranging from complete prohibition (i.e., compulsory removal of memorials) to allowing memorials to remain in place indefinitely (Churchill & Tay, 2008; Dickinson & Hoffmann, 2010). Although few jurisdictions explicitly permit memorials, many informally allow them and do not routinely dismantle them unless they are the subject of complaints (Churchill & Tay, 2008; Dickinson & Hoffmann, 2010). Notably, none of these policies are based on evidence about the impacts of memorials on road users. There are two especially relevant questions on the impact of roadside memorials: first, do they capture attention? Second, do they alter behavior in a way that impacts safety?

### Attentional capture

To our knowledge, there has been no previous research exploring the extent to which roadside memorials capture attention by using objective measures, such as eye movements. Several studies have asked drivers to self-report whether they consider memorials distracting, with mixed results. Many drivers believe memorials are potentially distracting but some drivers, especially young adults, believe they constitute a “positive distraction” (i.e., making drivers think more about road safety; Churchill & Tay, 2008; Hartig & Dunn, 1998; Tay, 2009). Advocates of roadside memorials argue that they are typically small and designed as a subtle place marker for grieving individuals, rather than trying to divert drivers’ attention away from the road (Collins & Rhine, 2003).

From a psychological perspective, it is plausible that even small memorials may capture drivers’ attention if they are visible from the roadway, because they are visually distinctive (e.g., white crosses and flowers pinned to a railing). Furthermore, they signal danger (“someone died here”) and threat-related stimuli have been found to preferentially capture attention (e.g., Öhman, Flykt, & Esteves, 2001). This so-called *threat superiority effect* has been found for both phylogenetic or evolutionary threats (e.g., angry faces, spiders, snakes) and ontogenetic or modern threats, such as weapons (Fox, Griggs, & Mouchlianitis, 2007), even though different brain areas are involved in processing biological versus manmade threats (Yang, Bellgowan, & Martin, 2012).

If a threat superiority effect occurs for roadside memorials, then in the presence of roadside memorials drivers should display a greater number of fixations and longer dwell times to the roadside area, away from the road. This diversion of eye movements could potentially negatively impact vehicle control, as extended glances away from the road are correlated with increased crash risk (Horrey & Wickens, 2007; Liang, Lee, & Yekhshatyan, 2012; Simons-Morton, Guo, Klauer, Ehsani, & Pradhan, 2014).

### Safety impacts

There is currently very little research examining the effects of roadside memorials on road user behavior and safety (Churchill & Tay, 2008; Tay, Churchill, & de Barros, 2011). Collins and Rhine (2003) reported that a “troubling number” of rear-end collisions in Arizona, USA involved a driver slowing down to look at a roadside memorial, but did not provide quantitative data to support this claim. Tay and colleagues (Tay, 2009; Tay et al., 2011) conducted studies in which they evaluated short-term effects of roadside memorials by constructing fake memorials, and long-term effects by comparing a real memorial site with two control sites. They found that placing a memorial near an intersection reduced red-light violations by an estimated 28.7% (Tay, 2009), but placing a memorial on a freeway did not influence passing traffic speeds (Tay et al., 2011). Some drivers self-report that they slow down or otherwise become more cautious after seeing a memorial, but the proportion of drivers who report this behavior varies greatly, from 7 to 8% in a sample of young Canadian drivers (Churchill & Tay, 2008) to half of all survey respondents in rural Australia (Hartig & Dunn, 1998). This suggests the effect of roadside memorials may be context-specific, although the evidence is limited.

### The current study

The current study was designed to examine the potential effects of roadside memorials on drivers, specifically by examining how the presence of memorials in road scenes affects observers’ eye movements and risk perception. Participants viewed videos of road scenes, some of which contained roadside memorials. It was predicted that participants would make more fixations to the left roadside (i.e., the side on which the memorial appeared), and would fixate on the roadside for a longer duration when a memorial was present. Participants were asked to indicate what speed they would drive (preferred travel speed), and how safe or risky it would be to drive on the road (risk ratings). Based on previous research, participants were expected to give higher risk ratings to roads that feature roadside memorials. Logically, higher risk ratings should be associated with adopting lower travel speeds (as in Charlton, Starkey, Perrone, & Isler, 2014); however, given the inconsistent findings from previous research on roadside memorials, there may be no significant difference in speeds. As a control, participants were also asked to report the posted speed limit for each road, as this should not be affected by the presence of memorials.

## Method

### Participants

A total of 40 observers (29 female and 11 male) aged 20–44 years (M = 30.7, SD = 6.5) were recruited and offered a gift card (AUD$20/h) as compensation. All were fluent in English and had normal or corrected-to-normal visual acuity. Participants were required to hold a current valid open (unrestricted) driver’s license and drive at least once a week. To attain an open driver’s license in Queensland, drivers must accrue 100 h of supervised practice as a learner, and pass practical and theoretical tests, which grants them a provisional license to be held for 3 years. This then allows them to drive unaccompanied with some restrictions (e.g., stricter blood alcohol limits). The inclusion criteria imposed therefore ensured all participants were experienced, regular drivers.

### Design

The experiment featured a repeated-measures design with a single factor (roadside object type: memorial, none/control, traffic cone).

### Apparatus

Visual stimuli were presented on a 22″ monitor with 1920 × 1080 resolution. Viewing distance was approximately 60 cm, but head position was not fixed, to allow naturalistic observation. Eye movements were tracked using a SensoMotoric Instruments (SMI) REDn eye-tracker, which tracks eye movements at 60 Hz. Stimulus presentation and data acquisition were controlled using SMI Experiment Center.

### Stimuli

Stimuli were 40 short video clips of daytime road scenes filmed from the driver’s perspective using a GoPro digital video camera. This comprised 10 videos of roads with roadside memorials and 30 videos of roads without roadside memorials. Among the 30 videos without memorials, 20 were matched clips (of which 10 included an added non-memorial roadside object), and 10 were “filler” clips. Filler clips were included to lower the relative prevalence of memorials in the stimulus set and were not included in the analyses.

Clips were selected and classified by three individuals (both authors plus one independent research assistant) to ensure that each clip unambiguously fit in a single category (i.e., memorial or non-memorial), and to code key events (e.g., weather conditions, traffic volume, presence of vulnerable road users, presence of hazards or potential hazards). Location, posted speed limit, and date/time of recording were noted for each clip.

Clips with memorials present were filmed by driving a passenger vehicle on real public roads with genuine roadside memorials present. Each clip was 23–44 s long, including at least 10 s of footage before the roadside memorial became visible (M = 17 s, SD = 6) and at least 2 s after it was passed (M = 11 s, SD = 6).^1^ The memorials were visible for 1.3–5 s (M = 2.5, SD = 1.1); this duration varied because of travel speed, road geometry and visibility. Clips were only included if the roadside memorial was a white cross located on the left side of the road. Only left-located memorials were included because this is where most roadside memorials in Australia are situated (as Australians drive on the left), and because drivers will naturally differ in the amount of time they fixate on the near versus the far side of the road, so it was necessary to control the side of the road on which the memorials appear. Only white crosses were included because this was the most common form of memorial, meaning they were visually similar between clips and were easily visible in the video. Other types of memorials, such as flowers or banners/plaques, were initially filmed as potential stimuli, but were not used because they were not obviously recognizable as memorials in video footage.

Matched clips were selected such that for each clip with a memorial present, there were two matching clips without memorials filmed on the same road or a similar road (see Additional file 1). Where it was not possible to film matched clips on the same road (e.g., because the road is too short, or because the road sections with memorials differ markedly from sections without memorials), matched clips were filmed on a nearby road that was as similar as possible in terms of all relevant features, such as number of lanes, presence/width of shoulder, horizontal and vertical curvature, roadside features (e.g. foliage, types of buildings, bridges) and travel speed.

The first set of 10 matched clips were the “control” condition and were matched as closely as possible to the memorial clips, except that they contained no memorials: 9 clips were filmed on the same road as the corresponding memorial clip, and most were filmed on the same day during the same filming session. Where it was necessary to film additional footage on a different day, it was filmed on a day with similar weather and traffic conditions.

A second set of 10 matched clips was filmed, with an added roadside object. This condition was added to assist interpretability: if the initial memorial-present versus memorial-absent comparison resulted in a significant difference, especially in glance behavior, it could be simply because the manipulation involved comparing something (memorials) with nothing (no memorials). To address this, we added a third condition, in which a traffic cone was placed on the left side of the road. A standard orange traffic cone was used because it is an object that is often found by the roadside, can also signify hazards and is a similar size to many of the cross memorials. Although the two target objects may differ in physical saliency, previous research has demonstrated that visual saliency does not influence or predict eye movements when viewing real-world scenes, despite being highly influential in basic visual search arrays (Henderson, Brockmole, Castelhano, & Mack, 2007). The same traffic cone was used in all videos containing the traffic cone and was specifically placed at an appropriate roadside location by a researcher before filming the video clips. Memorial and traffic cone clips were filmed on the same roads when filming on high-speed roads (80–110 km/h). It was not possible (due to road length and features) to film the traffic cone clips on the same road when filming on moderate-speed roads (60–70 km/h), so they were filmed on roads that had similar features.

### Procedure

Participants were tested individually in a quiet, dark room. Prior to the experiment, participants were informed that the research was examining risk perception and eye movements when viewing different types of roads (with no mention of roadside memorials). They were instructed to view each clip and to make three judgements: what travel speed they would adopt on the road (in kilometers/hour); what they believe is the posted speed limit (in kilometers/hour); and how risky or safe it would be to drive on this road (rated on an 11-point scale where 0 represents very safe and 10 represents very risky). Participants were asked to verbally explain their reasons for these judgements. The explanations were recorded using a digital audio recorder and transcribed verbatim for analysis.

Participants completed three practice trials before the experiment. During the practice block, eye movements were not tracked and participants had an opportunity to clarify instructions and task requirements as necessary. None of the practice clips contained memorials.

The eye-tracker was calibrated at the beginning of the experimental block using a 9-point calibration grid and re-calibrated every eight trials to ensure accurate gaze tracking was maintained. Calibration accuracy was validated using a 4-point grid, and only accepted if the average error was <  0.5 °.

Following the eye-tracking data collection, participants completed a brief questionnaire that asked them what they thought the study was about (i.e., to assess whether they realized the researchers were interested in roadside memorials). Finally, they were asked a series of questions about roadside memorials, including how frequently they encounter them, whether they are aware of adjusting their behavior around roadside memorials (e.g., reducing speed, increasing following distance), and whether they believe memorials should be permitted.

### Data analysis

Power analysis using G*Power (Faul, Erdfelder, Buchner, & Lang, 2009) indicates that a sample size of 40 would have sensitivity to detect an effect size of *d*_z_ = 0.53 with power of 0.95 in a pairwise within-subjects comparison. This would be considered a medium to large effect. It is likely that experimentally induced effects would be larger than any real-world changes in behavior, as real-world travel speeds are constrained and influenced by a multitude of interacting factors (e.g., other vehicles, weather), whereas the current study involves manipulating a single factor. Further, as the results could have relevance to real-world policy, it is appropriate to consider effects that are large enough to produce a meaningful impact under complex real-world conditions.

Statistical analyses were conducted in SPSS. Quantitative variables relating to eye movements and risk perception were analyzed using generalized estimating equations (GEE; Liang & Zeger, 1986), which is an extension of the general linear model that can be used for analysis of continuous, scale and binary variables that involve repeated measures with possible correlations. This approach was used instead of simpler repeated measures such as *t* tests or analysis of variance (ANOVA) (which were planned), which require averaging values within a condition, because there was large variability between trials, especially in speed data. Therefore, the analysis focused on individual trials, rather than condition averages. The models specified an exchangeable correlation matrix. A binary logistic generalized estimating equation (GEE) was used for binary variables (e.g., probability of fixation), a negative binomial GEE was used for count variables (e.g., number of fixations) and a linear GEE was used for continuous variables. Statistical significance was assessed using an alpha level of .05. Descriptive statistics were reported for the post-experiment questionnaire.

Dynamic areas of interest (AOIs) were coded during this period, including the road, left roadside, right roadside (if visible within frame), and the horizon/sky. AOIs were defined within the video clips, so that they were applied consistently across all observers. The left roadside AOI was the focus of analysis, as this was the area where roadside memorials and traffic cones were placed. For the memorial and traffic cone clips, a custom “target object” AOI was added, which was valid only for the temporal period in which the memorial/cone was visible, to assess whether participants directly fixated on it. The SMI software uses dispersion-based algorithms to detect fixations because the system’s temporal resolution is 60 Hz. The default parameters were applied, which were minimum fixation duration of 80 ms with maximum dispersion of 2.00 °. Blinks, fixations outside of any AOI and periods where tracking was lost were treated as missing data.

Three aspects of eye movements were compared between the memorial video clips and the matched non-memorial clips: probability of fixating roadside AOI (i.e., number of trials on which the left roadside area is fixated); number of fixations on roadside (average number of fixations per trial in the roadside area); and total fixation on roadside (average dwell time on roadside area, in milliseconds). The left roadside AOI analysis focused on 1314 ms of each clip, comprising the last 1280 ms when the memorial/traffic cone was visible, plus an additional 17-ms buffer before/after this period to account for the eye-tracker recording in 16.7-ms frames. This duration was selected because all memorials/cones were visible for at least 1280 ms. For control clips, the time period was matched to the period used for the memorial clips. The object AOI analysis focused on the full period that the object (memorial or cone) was visible; because this varied between trials, fixation duration was converted into a percentage of the visible time. Three other quantitative variables were compared between the memorial and non-memorial clips, specifically, posted speed limit, self-nominated travel speed and risk rating.

Verbal comments were coded to assess which aspects of the road participants considered when making their speed and risk ratings; specifically, whether they commented on the roadside memorial, if present. This involved coding concepts related to memorials (e.g., cross) and accidents (e.g., “someone died there”). These were used to assist in interpretation of the statistical analyses; for instance, if the presence of memorials affected eye movements but not risk ratings, this could be because participants are not considering the presence of memorials as a relevant factor when judging the safety of the road.

## Results

### Sample characteristics

Participants had held their license an average of 13.6 years (SD = 6.4, range 3.5–28.2) and reported driving an average of 302 km (SD = 255) or 8.8 h (SD = 6.4) per week. Most participants had not received any fines (80%) or been involved in any crashes (77.5%) in the preceding 12 months. Six participants reported having been involved in a property-damage-only crash as a driver within the past 12 months, with three others having been involved in a property-damage-crash as a passenger. No participants had experienced a crash that resulted in injury of any parties within the past 12 months.

### Verbal commentary

Most participants noticed at least one of the memorials: 26 participants (65%) commented on at least one memorial while viewing the clips. Two additional participants mentioned afterwards that they had seen the memorials, even though they did not comment on them while viewing the clips. However, most only explicitly commented on 1 (*n* = 9, 23%) or 2 (*n* = 9) of the 10 memorials, with the largest number of memorials noted being 6. In total, there were 61 instances (15% of the 400 memorial-present trials across all 40 participants) in which participants commented on the memorials. The comments varied: some simply mentioned the cross (e.g., “there’s a cross on the left hand side”), some noted that it signified a crash site (e.g., “There’s a cross so obviously someone has had an accident on this road”), whereas others simply mentioned that someone had died without mentioning the memorial itself (e.g., “someone’s died there”). Among participants who mentioned memorials, only half (*n* = 13) explicitly mentioned that they factored a memorial into one of their risk ratings (e.g., “From seeing those crosses it makes me think it would be really risky, maybe people fall asleep if it’s a long road?”). One participant repeatedly reported finding the sight of memorials quite distressing:

“Someone died back there. I hate those signs on the side of the road, I find that very traumatising and now I’m going to think about it for the whole rest of the drive down the highway, that someone died there. That’s distracting to me.” (Clip 7)

“Look there’s another one of those crosses on the side of the road. Are they, I just don’t get it. I think there should be a law that stops people having crosses on the side of the road, it’s dangerous.” (Clip 4)

In contrast, only 8 (20%) participants commented on the traffic cones. All 8 commented on only 1–2 of the 10 cones presented, meaning that participants commented on < 3% of traffic cones, and only 1 person suggested the cone might signify increased risk (“there was a traffic cone at one point in front of the bridge, so perhaps there’s damage to that area”).

### Eye movements

#### Probability of fixating left roadside

Participants fixated the left roadside AOI during 57% of all trials (when considering only the 1314-ms interest period). There was a significant main effect of experimental condition on fixation probability, χ^2^(2) = 18.92, *p* < .001. Participants were significantly less likely to fixate the left roadside area in the control condition compared with the memorial condition, χ^2^(1) = 17.49, *p* < .001, odds ratio (OR) = 0.67, 95% confidence interval (CI) for OR = 0.56, 0.81. There was no significant difference between the memorial and traffic cone conditions, χ^2^(1) = 0.61, *p* = .436, OR = 1.10, 95% CI for OR = 0.87, 1.39.

#### Number of roadside fixations

Participants made 0–6 fixations on the left roadside area during the 1314-ms interest period. Number of fixations significantly differed between experimental conditions, χ^2^(2) = 63.82, *p* < .001. Compared with the memorials-present condition, participants made significantly fewer fixations in the control condition, χ^2^(1) = 63.81, *p* < .001, B = −  0.5, SE = 0.07, but not the traffic cone condition, χ^2^(1) = 2.51, *p* = .113, B = −  0.1, SE = 0.06.

#### Dwell time on roadside area

**Fig. 1:**
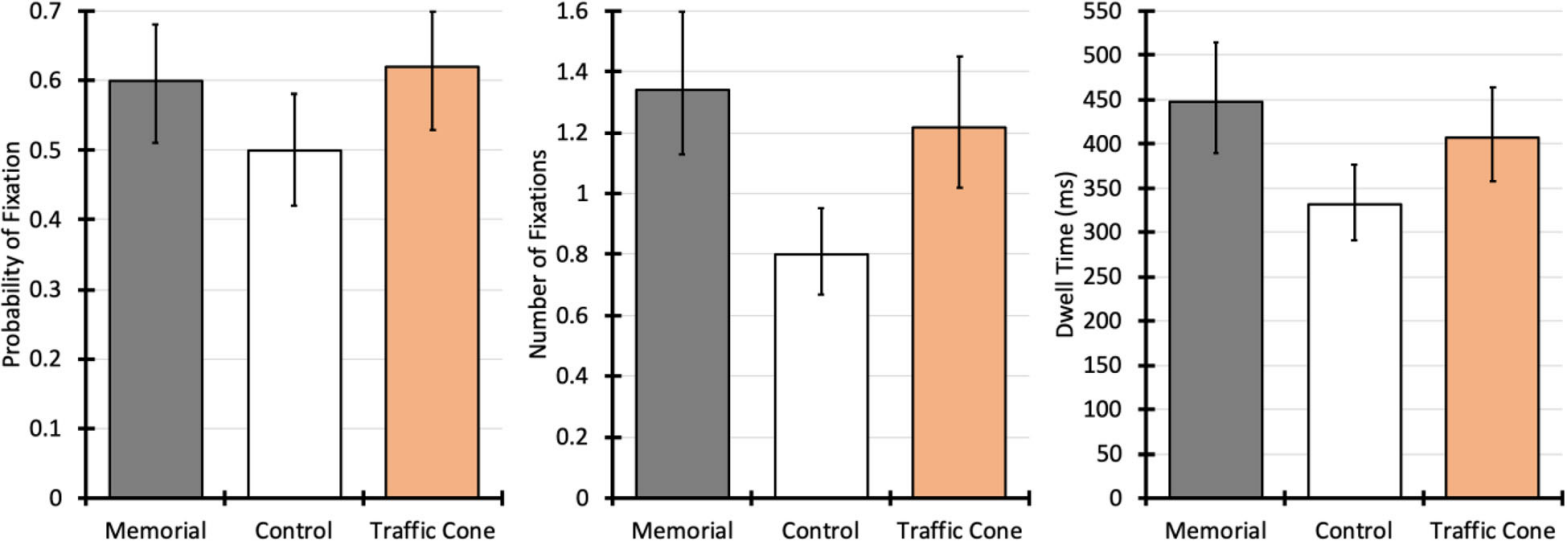
Eye movements to the left roadside area during the 1314-ms interest period. Error bars represent 95% confidence intervals

Dwell time was calculated as the total of all fixations on the left roadside during the 1314-ms interest period. There was a main effect of experimental condition, χ^2^(2) = 25.61, *p* < .001, as shown in Fig. 1. Dwell time was significantly shorter in the control condition, χ^2^(1) = 25.38, *p* < .001, B = −  0.30, SE = 0.06, but not the traffic cone condition, χ^2^(1) = 3.04, *p* = .081, B = −  0.10, SE = 0.05, compared with the memorial condition.

#### Target fixations

**Fig. 2:**
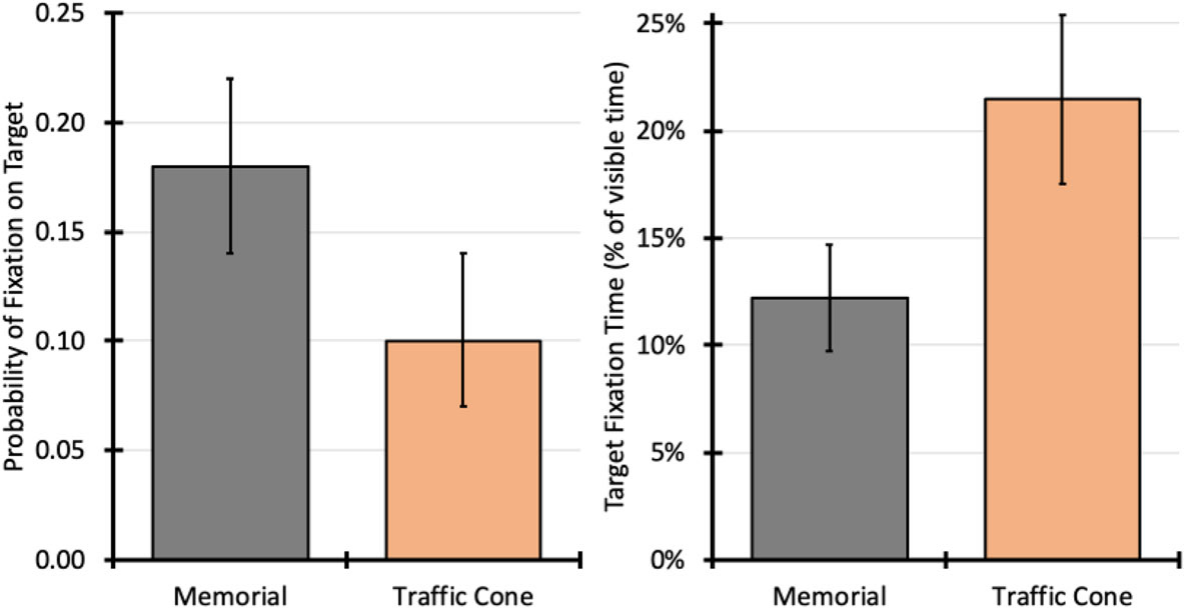
Probability of fixating on the roadside target object (memorial or traffic cone; left panel) and total fixation time (right panel) as a percentage of visible time. Error bars represent 95% confidence intervals

Target fixations were defined as fixations directly on the area of the memorial or traffic cone. Control trials were therefore not included in this analysis. Data were taken from the entire period that the target was visible (which varied between trials), so dwell time was calculated as a percentage of visible time, rather than absolute time in milliseconds. Participants were significantly less likely to fixate the traffic cones (10% fixated, 95% CI 7, 14) compared with the memorials (18% fixated, 95% CI 14, 22), χ^2^(1) = 12.74, *p* < .001, OR = 0.49, 95% CI for OR 0.33, 0.73, and made significantly fewer fixations on the traffic cones, χ^2^(1) = 10.33, *p* = .001, B = −  0.7, SE = 0.2. However, participants spent significantly less time fixating on memorials (12.2% of visible time, 95% CI 9.7, 14.7) compared with traffic cones (21.5% of visible time, 95% CI 17.5%, 25.4), χ^2^(1) = 17.04, *p* < .001, B = 0.09, SE = 0.02, see Fig. 2. Note that when analyzing absolute fixation time, instead of percentages, this trend remained (traffic cone, M = 482 ms, 95% CI 403, 577; memorial, M = 408, 95% CI 331, 50]) but was not statistically significant.

Interestingly, the trials on which participants fixated the memorial were not necessarily trials during which they mentioned the memorial. Participants both fixated and mentioned the memorial in 8% of trials in which a memorial was presented, but in 10% of trials they fixated the memorial without mentioning it and on 7% they mentioned it despite not directly fixating on it. Participants fixated on and mentioned the traffic cone in 1% of trials, fixated on it without mentioning it in 8.75% of trials, and mentioned it without fixating on it in 1.75% of trials. Because participants were not explicitly asked about whether they noticed these objects in specific trials, the fact that 8–10% fixated on but did not comment on these objects does not necessarily mean that they “looked but failed to see”; rather, they may not have considered these objects relevant to their interpretation of the road scene.

### Speed

#### Posted speed judgements

Because speed questions were answered verbally, participants could give answers that were not specific integers. The following rules were applied for the actual speed limit question: “X or less” was coded as X (i.e., “50 or less” = 50); “less than X” was coded as the next valid speed limit under X (i.e., “less than 50” = 40); and if multiple options were suggested (e.g., “100 or 110”), the higher speed was used unless they explicitly indicated that the lower speed was more likely.

**Fig. 3:**
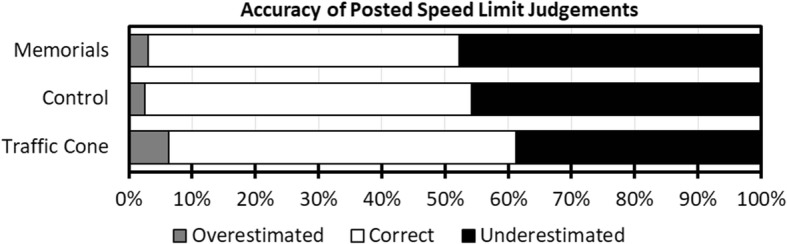
Accuracy of posted speed limit judgements by experimental condition

Answers were then matched against the actual speed limit, to assess whether participants’ estimates were accurate, overestimated, or underestimated. This analysis revealed that participants only correctly identified the posted speed limit in 52% of trials overall and were more likely to underestimate the speed limit (44%) than overestimate it (4%). Ordinal logistic GEE indicated that estimate category (overestimate, correct, underestimate) varied significantly with experimental condition, χ^2^(2) = 24.35, *p* < .001. Pairwise comparisons revealed that there was no significant difference between the memorial and control clips, χ^2^(1) = 0.72, *p* = .397, but there was a significant difference when comparing the memorial and traffic cone clips, χ^2^(1) = 23.92, *p* < .001. Participants were less likely to underestimate and more likely to overestimate the speed limit in the traffic cone clips (see Fig. 3). As noted in the method, memorial and control clips were nearly all filmed on the same roads, whereas several of the traffic cone clips had to be filmed on different roads. When excluding clips that were filmed on different roads, the trend remained but the model effect was no longer statistically significant, χ^2^(2) = 5.62, *p* = .060, suggesting this effect was an artifact of road features and not attributable to the presence or absence of memorials.

#### Likely driving speed judgements

For the question on what speed they would likely drive, responses were dealt with in a similar manner as for actual speed. If they indicated they would drive at the speed limit, the value they gave for actual speed limit was used. If they specified a range, the midpoint was used (e.g., “60–70” = 65). If they said they would drive “just over/under” a given speed, this was coded as ±3 km/h from the stated speed (e.g., “just over 100” = 103).

**Fig. 4:**
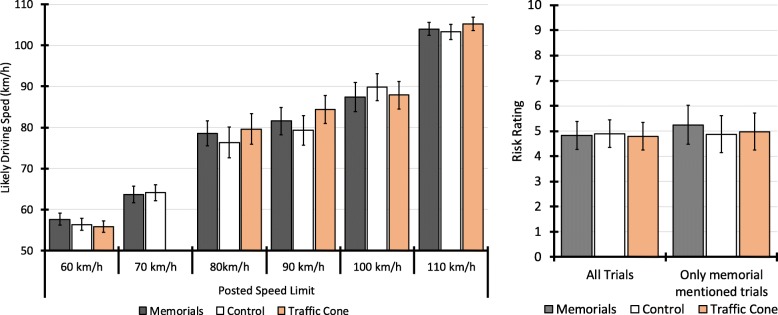
Participants’ self-nominated likely travel speeds (left panel) and risk ratings (right panel) by experimental condition. Error bars represent 95% confidence intervals. Note: there was only one clip with a 70 km/h official limit for the memorials and control conditions, and none for the traffic-cone condition as the road with the most similar road in terms of features had a 60 km/h limit

Initial analysis including all trials indicated no effect of experimental condition on self-nominated driving speeds, χ^2^(2) = 3.59, *p* = .166. A limitation of this analysis is that the posted speed limits for the roads depicted in the clips varied from 60 km/h to 110 km/h, meaning there was greater variation within conditions than between, and behavioral patterns may differ for high-speed versus moderate-speed roads. When road speed limit was added to the model as a factor, there was a significant effect of experimental condition, χ^2^(2) = 8.09, *p* = .017, but also a significant effect of road-speed zone, χ^2^(5) = 3854.77, *p* < .0011, and a significant interaction between experimental condition and speed zone, χ^2^(9) = 37.14, *p* < .001 (see Fig. 4). To interpret the interaction, separate analyses were therefore conducted for each speed zone.

For videos filmed on 110 km/h roads, there was a significant main effect of condition, χ^2^(2) = 28.99, *p* < .001. Participants’ self-nominated travel speeds were significant slower in the memorial condition (M = 104.0 km/h, 95% CI 102.5, 105.6) compared with the traffic cone condition (M = 105.2, 95% CI 103.6, 106.8), χ^2^(1) = 11.38, *p* = .001, B = 1.2, SE = 0.04. This effect is very small but was statistically significant due to range restriction (most, if not all, participants would be familiar with the 110 km/h road - the Bruce Highway, the major state highway - and would know the speed limit). There was no significant difference between the memorial and control conditions, χ^2^(1) = 2.02, *p* = .155, B = −  0.7, SE = 0.5.

For the clips filmed on 60 km/h roads, there was also a significant main effect of condition, χ^2^(2) = 8.85, *p* = .012, but the direction of the effect was reversed. Participants’ self-nominated travel speeds were significantly *faster* for clips containing memorials (M = 57.6, 95% CI 56.1, 59.]) compared with those containing traffic cones (M = 55.8, 95% CI 54.4, 57.2), χ^2^(1) = 8.82, *p* = .003, B = − 1.8, SE = 0.6. There was no significant difference between the memorial and control conditions, χ^2^(1) = 2.86, *p* = .091, B = − 1.3, SE = 0.8.

The analyses did not reveal significant main effects of condition for clips featuring roads with speed limits of 70 km/h (χ^2^(2) = 0.38, *p* = .535), 80 km/h (χ^2^(2) = 3.49, *p* = .175) or 100 km/h (χ^2^(2) = 3.64, *p* = .162). For the 90 km/h clips, there was a significant main effect of condition, χ^2^(2) = 6.28, *p* = .043. However, pairwise comparisons were not statistically significant when comparing the memorials condition to either the control condition, χ^2^(1) = 1.75, *p* = .186, B = − 2.3, SE = 1.7, or the traffic cone condition, χ^2^(1) = 2.66, *p* = .103, B = 2.8, SE = 1.7. In other words, the significant main effect reflected differences between the control and traffic cone conditions.

### Risk ratings

There were weak, negative, but statistically significant (*p* < .001) correlations between risk ratings and both the posted speed limit, *r* (1159) = − .18, 95% CI − .23, − .12, and participants’ likely driving speed, *r* (1159) = − .10, 95% CI − .17, − .05. To account for the repeated measurements (i.e., each participant made multiple ratings), the correlations were calculated using the rmcorr package in R (Bakdash & Marusich, 2017). Negative correlation indicates that participants rate higher-speed roads as less risky than lower-speed roads. Although this may seem counterintuitive, the highest-speed roads (110 km/h) lacked many of the features that participants mentioned when explaining risk, such as intersections, curves, pedestrians, school children and dense traffic. Because the correlation between speed limit and risk rating was so weak, the risk ratings were analyzed for all trials, collapsing across speed limits.

When considering the full sample of all trials, there was no significant effect of experimental condition on risk ratings, χ^2^(2) = 3.83, *p* = .148. When considering only trials in which the participant explicitly mentioned the memorial (and the matched comparison clips), the main effect of experimental condition was still not statistically significant, χ^2^(2) = 4.32, *p* = .115. Although this analysis is underpowered as it is based on a total of 183 trials (i.e., the 61 trials in which participants explicitly mentioned the memorial, and the matching 61 control and 61 traffic cone trials), as shown in Fig. 4, risk ratings were very similar between conditions.

### Opinions on roadside memorials

When asked to describe what they thought the study was about, after having participated, no participants mentioned roadside memorials. All mentioned other relevant concepts, such as visual attention, hazard perception, risk perception and risk awareness, speed limits and eye movements.

**Fig. 5:**
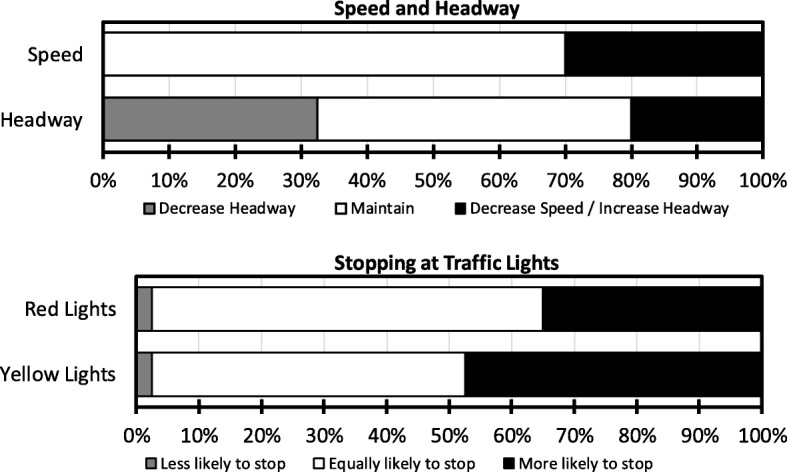
Self-reported driver behavior when driving past roadside memorials. Note that white shaded areas represent no behavioral change in response to memorials; black represents those reporting positive safety-related behaviors (slowing, stopping) and gray indicates less safe behaviors

All participants indicated that they had noticed a roadside memorial while driving, and encountered them at least once a month, with over two thirds indicating that they encountered them at least once a week (22.5% daily, 20% 3–5 days/week, 27.5% 1–2 days/week). Figure 5 depicts self-reported behavior driving past roadside memorials. In general, most respondents indicated they would not behave differently when passing memorials, but a sizeable minority indicated they would adopt safer behaviors, such as decreasing speed (30%) and increasing headway (20%) or being more likely to stop at red (35%) and yellow lights (48%). Approximately one third said they would *decrease* headway, which was explicitly defined as “following distance”. The latter result seems counterintuitive, and probably reflects participants not fully understanding or thinking about the concept (e.g., just assuming “decreasing” meant adopting a safer behavior for both speed and headway).

When asked to describe what they thought about when driving past memorials, the most common themes were wondering how the accident occurred (43%), especially if the road did not seem obviously hazardous; the need to take care because it is a dangerous area or driving itself is inherently dangerous (35%); the fact that someone died (33%); feelings of sadness (33%) and thoughts about the loved ones of the deceased (25%). Several participants also made comments about speed and/or the need to slow down (18%), with a smaller proportion commenting on characteristics of the memorial itself (13%), such as flowers or “ghost bikes” or wondering who it was that died (13%).

Nearly all participants (95%) indicated that roadside memorials should be permitted. The two participants who thought they should be prohibited both suggested they could be distracting. Interestingly, the participant who repeatedly commented negatively about memorials while viewing the clips later indicated in the post-experiment questionnaire that they should be permitted, because they help remind others how dangerous driving can be. Several participants who thought memorials should be allowed also noted that they could potentially distract drivers (11%), for example, if the memorials were large/salient or if drivers were especially sensitive, but they either believed that the benefits outweighed this potential risk or “it’s up to individual drivers to ignore them”. Overwhelmingly, the most common theme in the comments by those who endorsed roadside memorials was that they were an effective safety message (76%) to signal danger and remind drivers to take care: “They are a reminder for other road users that that section of road can be dangerous enough for a fatal accident, even if it appears safe.” One person declared they were “essential!” for this reason, while another noted “if … the roadsides become too full of the memorials well that speaks for itself”. Other recurring themes were that memorials were helpful to family and friends of the deceased (29%) and that they did not pose a danger or distraction (16%). A few of these participants specifically mentioned that memorials were no more distracting than billboards. However, several people mentioned the need to regulate or limit the scope of memorials (21%); for example, ensuring they were safely located, small and not too eye-catching. One person suggested there should be a time limit, particularly so that drivers (especially emergency services personnel) do not have to be repeatedly reminded of the fatality.

## Discussion

This study was designed to systematically explore the impact of roadside memorials on drivers’ attention and risk perception. In general, the findings suggest that roadside memorials capture drivers’ attention at least some of the time, but do not necessarily have a large or meaningful impact on safety-related behaviors - despite the fact that many drivers believe that memorials can improve safety.

Analysis of eye tracking data provides evidence that roadside memorials can capture attention. Participants were more likely to look at the left roadside area and spent longer fixating on it when memorials were present, compared with the memorial-absent control condition. This effect seems to be partly because there was a roadside object to look at, because there was no significant difference in glance behavior to the left roadside when comparing the memorials condition with the traffic-cone condition. Nevertheless, participants were significantly more likely to fixate directly on the memorials themselves compared with traffic cones, suggesting that memorials are slightly more likely to capture attention than some other roadside objects. Although these effects were statistically significant, they were small in magnitude and the total fixation time on memorials averaged approximately 400 ms. Such short glances away from the forward roadway are common and are not considered unsafe (Horrey & Wickens, 2007; Liang et al., 2012). Combining data from eye movements and verbal comments suggest that at least 25% of the memorials featured in the experiment captured participants’ attention, and consistent with this most participants reported noticing memorials regularly while driving.

Participants were nearly all supportive of roadside memorials, with most believing that they provide a useful safety message to remind drivers to take care and signal that a specific road is especially dangerous. This is consistent with previous research on attitudes toward memorials (Churchill & Tay, 2008; Hartig & Dunn, 1998; Tay, 2009), although our sample were even more supportive. Despite their belief that memorials convey an important safety message, the presence of memorials did not systematically alter perceived risk. The findings on travel speed were mixed; in the post-experiment questionnaire, most participants indicated that seeing a memorial would not make them alter their travel speed or stopping behavior at traffic lights. Only 30% indicated they would reduce their speed, which is smaller than the proportions reported in previous Australian research (Hartig & Dunn, 1998). The post-experiment questionnaire likely overestimates behavioral change, as participants may be prompted by the question to think of occasions when they have changed their behavior, even if they do not always do that.

When viewing videos of road scenes and asked to select a speed, without being explicitly asked to take account of the memorials, there was no clear impact of memorials on self-nominated travel speed. Specifically, there were no significant differences in self-nominated travel speeds between the memorial and control conditions, suggesting that even though many drivers say they will slow down, they do not necessarily actually adjust their speed. This could be because they do not always notice memorials, or because they typically drive at or below the speed limit, so they do not think further speed reductions are required. There were some significant differences between the memorial and traffic-cone conditions, but the effects were small, inconsistent (i.e., travelling slower in 110 km/h zones but faster in 60 km/h zones) and most likely an artifact of the stimuli being filmed on different sections of the road with subtly varying features, despite our efforts to match the stimuli as closely as feasible. This highlights a fundamental limitation of conducting experimental research with naturalistic stimuli; researchers must consider the potential for hidden confounders. In the current study, having two different control conditions helped interpret significant differences, but also made it more difficult to precisely match the stimuli. It is notable that although there were several significant effects, in all cases only one of the two comparison conditions differed significantly from the memorial condition. This suggests that the effects are either not solely attributable to the presence of memorials or are not sufficiently large to be meaningful.

Overall, our data do not provide any evidence that roadside memorials meaningfully improve road safety: their presence did not impact risk perception and had little to no impact on drivers’ speed choices. The memorials used in our current study did not induce any effects that would have a clearly negative impact on safety either, but notably a small number of participants expressed strong negative reactions to or opinions on memorials. Many drivers reported emotional reactions when passing memorials, ranging from brief sadness (e.g., “how sad”) to fairly intense discomfort (e.g., during post-experiment debriefing, asking the researcher if the study results could be used to ban memorials). Despite this, most of the memorials shown did not evoke any measurable change in behavior at all - specifically, 75% of the memorials shown were neither fixated on nor commented on by participants. When the memorials were noticed, participants made at most a few short glances toward the memorial. This implies that memorials of the type shown - white crosses - are not unduly distracting for drivers in general, even though a small proportion of drivers find them distressing or inappropriate and think they should not be allowed.

One issue worth noting is that the stimuli were filmed on local roads, many of which are distinctively recognizable and likely familiar to participants; specifically, the Bruce Highway, Sunshine Motorway and Kawana Way. In other research that explicitly assessed location familiarity, we have found that 86–89% of participants recognize these specific roads (Beanland & Wynne, 2019). The impact of familiarity on hazard perception is not straightforward; some studies conclude drivers are better at hazard perception on familiar roads (Thompson & Sabik, 2018), whereas others find they are less attentive (Young, Mackenzie, Davies, & Crundall, 2018). In the current study, familiarity with the roads used as stimuli could have lessened the main effect of memorial presence, as participants’ evaluations of the roads may be influenced by their pre-existing knowledge of that specific road. Recognition of the road may activate a schema; for example, “the Bruce Highway is a dangerous road”. Participants who are unfamiliar with the road cannot activate the same type of schema must rely on identifying generic features to inform their risk assessment, such as high travel-speeds, horizontal or vertical curves, dense traffic or the presence of memorials. As such, one question for future research would be whether drivers are more affected by memorials on unfamiliar roads. This is speculative, but notably several participants reported wondering “what happened?” when they pass memorials, so it is plausible that location familiarity could lessen the impact (especially the shock value) of roadside memorials.

Many participants mentioned family and friends of the deceased when discussing memorials; this was even more common than comments about the deceased person. Many participants noted that memorials serve an important purpose for those who are grieving, although some expressed concern that people could put themselves in danger by being on the roadside to build, maintain and mourn at memorials. This raises an issue that has not been explored in previous research, which has mostly focused on vehicle-related behavior such as speeding and red-light running. Although this was mentioned by multiple participants, it is unknown how common it is for mourners to be standing at memorials and whether this is a genuine safety issue.

In conclusion, our data indicate there is not a strong evidence base for unilaterally banning roadside memorials. Although our data do not show clear negative or positive effects, it would be misleading to consider memorials as “safety neutral” because some participants reported quite strong negative emotional reactions or noted that others may have these types of reactions. Further, some participants commented that memorials were acceptable if they were “small” and not “extravagant” or “eye-catching”, suggesting that although they do not support prohibition of memorials, they felt they should be regulated somehow. Given the lack of conclusive evidence supporting a safety benefit or disbenefit, the decision to permit or prohibit roadside memorials could be based on other factors, such as aesthetics and whether the public support them (which in our sample they overwhelmingly did).

## Additional file

Additional file 1: Supplementary information: details of video clip stimuli. (PDF 654 kb)

## Footnotes

1 The preregistered plan specified ≥ 3 s before and after the memorial was visible, but for one clip this was not possible because the road contained two separate memorials close together. Thus, the clip was terminated before the second memorial became visible, which was 2 s after the first memorial was passed.
