# Supplementary material for: Effects of roadside memorials on drivers’ risk perception and eye movements
Source: Cogn Res Princ Implic. 2019 Aug 14;4:32. doi: 10.1186/s41235-019-0184-1 (PMC6694370; doi:10.1186/s41235-019-0184-1)
Supplement: Supplementary file 1 — Supplementary information: details of video clip stimuli. (PDF 654 kb) [file 41235_2019_184_MOESM1_ESM.pdf]

Supplementary Information: Details of Video Clip Stimuli

Clip Number	Clip ID	Condition	Length *(seconds)*	Target Visible	First Visible	Location (all Queensland, Australia)	Type	Speed limit (km/h)	Recorded
1	R01	Memorial	29.22	3 s	14.6 s	Old Cleveland Road, Coorparoo	Urban	60	17/05/18 07:55
	V01	Control	27.22	-	-				16/06/18 16:00
	T01	Object	32.00	2.4 s	14.2 s	Stafford Road, Stafford			24/06/18 16:30
2	R02	Memorial	23.22	3 s	15.0 s	Nicklin Way, Kawana	Urban	70	30/03/18 10:00
	V02	Control	26.26	-	-				30/03/18 10:00
	T02	Object	28.34	1.9 s	18.8 s	Caloundra Road, Caloundra		60	15/06/18 13:00
3	R03	Memorial	34.56	5 s	22.4 s	Bruce Highway, Beerburrum	Highway	110	04/05/18 13:30
	V03	Control	32.19	-	-				04/05/18 13:30
	T03	Object	29.23	2.9 s	12.2 s				15/06/18 11:00
4	R04	Memorial	44.33	2.4 s	19.0 s	Bruce Highway, Six Mile Creek	Highway	110	25/05/18 12:00
	V04	Control	33:32	-	-				25/05/18 12:00
	T04	Object	33.41	1.8 s	18.7 s				25/05/18 12:00
5	R05	Memorial	29.38	1.3 s	10.2 s	Bruce Highway, Coochin Creek	Highway	110	04/05/18 15:00
	V05	Control	21.37	-	-				04/05/18 15:00
	T05	Object	27.06	1.8 s	13.0 s				15/06/18 12:00
6	R06	Memorial	33.37	1.5 s	30.3 s	Bruce Highway, Mellum Creek	Highway	110	22/07/18 15:00
	V06	Control	31.24	-	-				15/06/18 10:00
	T06	Object	37.48	2 s	12.1 s				15/06/18 12:00
7	R07	Memorial	26.59	1.7 s	11.1 s	Sunshine Motorway, Mountain Creek	Highway	90	10/05/18 13:00
	V07	Control	21.52	-	-				15/06/18 15:00
	T07	Object	23.41	2 s	7.4 s				15/06/18 15:00

Cognitive Research: Principles and Implications
https://doi.org/10.1186/s41235-019-0184-1

Clip Number	Clip ID	Condition	Length *(seconds)*	Target Visible	First Visible	Location (all Queensland, Australia)	Type	Speed limit (km/h)	Recorded
8	R08	Memorial	25.38	3.8 s	13.7 s	Yandina Bli Bli Road, Bli Bli	Rural	80	10/05/18 13:20
	V08	Control	27.55	-	-				17/06/18 08:00
	T08	Object	25.22	1.3 s	9.8 s				17/06/18 08:00
9	R09	Memorial	33.45	1.5 s	20.0 s	State Route 70, Coolum	Motorway	100	17/06/18 09:00
	V09	Control	32.58	-	-				17/06/18 09:00
	T09	Object	34.18	1.5 s	17.1 s	State Route 70, Marcoola			17/06/18 09:00
10	R10	Memorial	31.05	2 s	16.5 s	South Pine Road, Everton Park	Urban	60	16/06/18 17:00
	V10	Control	26.55	-	-	Stafford Road, Stafford			24/06/18 17:15
	T10	Object	22.16	2.2 s	18.1 s	Webster Road, Stafford			24/06/18 16:30
